# Robust volcano plot: identification of differential metabolites in the presence of outliers

**DOI:** 10.1186/s12859-018-2117-2

**Published:** 2018-04-11

**Authors:** Nishith Kumar, Md. Aminul Hoque, Masahiro Sugimoto

**Affiliations:** 1 Department of Statistics, Rajshahi University, Rajshahi, Bangladesh; 2 Bioinformatics Lab, Department of Statistics, Bangabandhu Sheikh Mujibur Rahman Science and Technology University, Gopalganj, Bangladesh; 3 Health Promotion and Preemptive Medicine, Research and Development Center for Minimally Invasive Therapies, Tokyo Medical University, Shinjuku, Tokyo, 160-8402 Japan

**Keywords:** Metabolomics, Differential metabolites, Fold change, Classical volcano plot, Receiver operating characteristic (ROC) curve

## Abstract

**Background:**

The identification of differential metabolites in metabolomics is still a big challenge and plays a prominent role in metabolomics data analyses. Metabolomics datasets often contain outliers because of analytical, experimental, and biological ambiguity, but the currently available differential metabolite identification techniques are sensitive to outliers.

**Results:**

We propose a kernel weight based outlier-robust volcano plot for identifying differential metabolites from noisy metabolomics datasets. Two numerical experiments are used to evaluate the performance of the proposed technique against nine existing techniques, including the *t*-test and the Kruskal-Wallis test. Artificially generated data with outliers reveal that the proposed method results in a lower misclassification error rate and a greater area under the receiver operating characteristic curve compared with existing methods. An experimentally measured breast cancer dataset to which outliers were artificially added reveals that our proposed method produces only two non-overlapping differential metabolites whereas the other nine methods produced between seven and 57 non-overlapping differential metabolites.

**Conclusion:**

Our data analyses show that the performance of the proposed differential metabolite identification technique is better than that of existing methods. Thus, the proposed method can contribute to analysis of metabolomics data with outliers. The R package and user manual of the proposed method are available at https://github.com/nishithkumarpaul/Rvolcano.

**Electronic supplementary material:**

The online version of this article (10.1186/s12859-018-2117-2) contains supplementary material, which is available to authorized users.

## Background

In bioinformatics, molecular *omics* studies- like genomics, transcriptomics, proteomics and metabolomics are playing a prominent role in life sciences, health and biological research [1]. Among these approaches, metabolomics is frequently used to understand biological metabolic status, making a direct link between genotypes and phenotypes [2]. Many metabolomics-based biomarker discoveries have explored the key metabolites to discriminate between metabolic diseases, such as diabetes, cardiovascular diseases, and cancers [3]. The metabolites showing different concentrations among the given groups (e.g. healthy and disease subjects) is called as differential metabolites. Combinations of these metabolites can be used to identify subjects with a high risk of suffering from diabetes [4]. Thus, one of the most important tasks of metabolomics research is to identify a differential metabolite or a set of differential metabolites which have ability to differentiate patients with a disease from healthy subjects. The accurate identification of differential metabolites, or molecules that reflect a specific phenotype, is a cornerstone of many applications, such as predicting disease status and drug discovery [5–8].

To generate high-throughput metabolomics data, nuclear magnetic resonance (NMR) and hyphenated mass spectrometry (MS), such as gas chromatography-MS (GC-MS) and liquid chromatography-MS (LC-MS), are commonly used. These platforms can simultaneously identify and quantify hundreds of metabolites. All these analytical platforms can result in missing values in the observed data and outliers, which are caused by various reasons including analytical, experimental, and human errors, low quality measurements, malfunctioning equipment, and overlapping signals [9–20]. Thus, subsequent metabolomics data analysis should consider the presence of these problems in the given data.

Four types of statistical procedure have primarily been used to identify differential metabolites: (i) classical parametric approaches, such as Student’s *t*-test [21], classical volcano plot (CVP) [22] and fold change rank ordering statistics (FCROS) [23], (ii) classical non-parametric approaches, such as significance analysis of microarrays (SAM) [24], and the Wilcoxon [25] and Kruskal-Wallis (K-W) [26] tests, (iii) Bayesian parametric approaches, such as Bayesian robust inference for differential gene expression (BRIDGE) [27], empirical Bayes methods for microarrays (EBarrays) [28], and linear models for microarrays (Limma) [29], and (iv) Bayesian non-parametric approaches [30, 31]. In classical procedures, differential metabolites are identified using *p*-values (significance levels) that are estimated based on the distribution of a test statistic or a permutation, whereas in Bayesian procedures, differential metabolites are identified using posterior probabilities. However, most of the aforementioned techniques are not robust against outliers [27, 32]. Thus, they may produce misleading results in the presence of outlying samples or irregular concentrations of metabolites. Moreover, outlying samples or irregular concentrations of metabolites may violate the normality assumption in metabolomics datasets. Several nonparametric approaches (Wilcoxon and K-W test) and some Bayesian approaches (BRIDGE and Robust limma) are robust against outliers; however, increases in the number of outliers in these techniques reduce the accuracy of differential metabolite identification. One of the easiest ways to overcome this problem is to delete the outlying metabolites or outlying samples from the dataset. However, the deleted metabolites may be important metabolites in some cases, while deleting samples and metabolites can make the dataset much smaller or even vanish.

Comparatively, CVP [22] is a good technique for identifying differential metabolites because it can control the false discovery rate [33]. The volcano plot is based on *p*-values from a *t*-test and fold-change (FC) values [34], both of which depend on classical location and scatter, and thus volcano plot is affected by outliers. Therefore, in this paper, we develop an outlier-robust volcano plot by unifying CVP and a kernel weight function to overcome the problem of outliers. The advantage of the proposed method compared to existing methods is that it performs considerably better in the presence of outliers. We introduced a kernel weight function, which plays a key role in the performance of the proposed method. Robust volcano plot ensures robustness by producing smaller weights for outlying observations from the kernel weight function. Appropriate selection of the tuning parameter for the kernel function also improves the performance of the proposed method, as discussed later.

Since metabolomics dataset frequently contains outliers and all of the existing differential metabolite identification techniques are more or less influenced by outliers; as a result, outliers reduce the accuracy of differential metabolite identification. Therefore, in this paper we develop a kernel weight based outlier-robust volcano plot for detecting differential metabolites from metabolomics datasets in the presence of outliers. To measure the performance of the proposed method in comparison with other techniques, we consider nine existing differential metabolite identification techniques: three classical parametric approaches (*t*-test, FCROS, CVP), three nonparametric approaches (Wilcoxon test, K-W test, SAM) and three Bayesian approaches (BRIDGE, Limma, EBarrays). We also evaluate the performance of the proposed method using both artificially generated and experimentally measured metabolomics datasets in the absence and presence of outliers. Every metabolite identification method has a specific cutoff and its choices are sensitive to determine the metabolite identification which has large effect on the statistical analyses. In this paper, the cutoff of t-test, SAM, Wilcoxon test and K-W test have been taken as Bonferroni corrected *p*-value < 0.05. According to Dembélé et al. [23] we declared those metabolites as differential whose f-value is close to 0 or 1 and if f-value is close to 0.5 we took those metabolites as non differential. For CVP, a metabolite was said to be differential if *p*-value < 0.05 and | log_2_ (fold-change) | > 1. For Bayesian approaches we took the cutoff of Bonferroni corrected posterior probabilities > 0.95.

## Methods

In this paper, a kernel weight based outlier-robust volcano plot is developed for detecting differential metabolites. To reduce the family wise error rate when comparing the performance of the proposed method with existing differential metabolite identification techniques, the *p*-values are adjusted using Bonferroni correction. The algorithm for outlier-robust volcano plot is given below.

### Outlier-robust volcano plot (proposed)

We extend volcano plot by introducing a kernel weight function behind CVP. Classical volcano plot identifies differential metabolites using the *t*-test and fold-change (FC) methods, and plots log_2_ (fold-change) on the X-axis against *-log*_10_ (*p*-value) from the *t*-test on the Y-axis. Because the *t*-statistic depends on mean and variance and fold-change depends on mean, CVP is heavily influenced by outliers. Therefore, we use the kernel weighted average and variance instead of the classical mean and variance in the *t*-statistic and fold-change functions, and also plot *log*_*2*_ fold-change on the X-axis and *-log*_10_ (*p*-value) from the *t*-test on the Y-axis. We refer to this procedure as robust volcano plot (RVP).

Let *X* = (*x*_*ij*_); *i = 1, 2, …, p* and *j = 1, 2, …, n*, be a metabolomics data matrix with *p* metabolites and *n* samples. The rows and columns of *X* represent the metabolites and samples, respectively. In metabolomics data analysis, differential metabolites are the metabolites that show different concentrations between two groups (disease and control) of samples in a metabolomics dataset. According to the control and disease groups, the dataset can be expressed as

$$ X=\left[\begin{array}{c}\overset{Control}{\overbrace{x_{11}\kern0.5em {x}_{12}\kern0.5em \cdots \kern0.5em {x}_{1{g}_1}}}\\ {}{x}_{21}\kern0.5em {x}_{22}\kern0.5em \cdots \kern0.5em {x}_{2{g}_1}\\ {}\begin{array}{cccc}\vdots \kern0.75em & \vdots \kern0.5em & \ddots & \vdots \end{array}\\ {}\begin{array}{cccc}{x}_{p1}& {x}_{p2}& \cdots & {x}_{pg_1}\end{array}\end{array}\kern0.5em \begin{array}{c}\overset{Disease}{\overbrace{\begin{array}{cccc}{x}_{1\left({g}_1+1\right)}& {x}_{1\left({g}_1+2\right)}& \cdots & {x}_{1n}\end{array}}}\\ {}\begin{array}{cccc}{x}_{2\left({g}_1+1\right)}& {x}_{2\left({g}_1+2\right)}& \cdots & {x}_{2n}\end{array}\\ {}\begin{array}{cccc}\vdots \kern1.5em & \vdots \kern1.5em & \ddots & \vdots \end{array}\\ {}\begin{array}{cccc}{x}_{p\left({g}_1+1\right)}& {x}_{p\left({g}_1+2\right)}& \cdots & {x}_{pn}\end{array}\end{array}\right], $$

where *g*_1_ is the number of subjects in the control group and (*n* − *g*_1_) is the number of subjects in the disease group. In CVP, *log*_*2*_(fold-change) and *-log*_10_(*p*-value) from the *t*-test are calculated as follows.

The *log*_*2*_ (fold-change) value for the *i*-th metabolite is

1 $$ {\log}_2\left({FC}_i\right)={\log}_2\left(\frac{{\overline{X}}_i^D}{{\overline{X}}_i^C}\right), $$

where ${\overline{X}}_i^D$ represents the average intensity of the *i*-th metabolite for the disease group and ${\overline{X}}_i^C$ represents the average intensity of the *i*-th metabolite for the control group.

The *t*-statistic for testing the hypothesis that the *i*-th metabolite is not differential, i.e.

$$ {H}_0:\kern0.75em {\mu}_i^C={\mu}_i^D\kern1.5em \mathrm{against}\kern1.5em {H}_1:\kern1em {\mu}_i^C\ne {\mu}_i^D, $$

for *σ*_*iC*_^2^ = *σ*_*iD*_^2^ is

2 $$ t=\frac{{\overline{X}}_i^C-{\overline{X}}_i^D}{\sqrt{S_i^2\left(\frac{1}{g_1}+\frac{1}{n-{g}_1}\right)}} $$

where

$$ {\displaystyle \begin{array}{l}{\overline{X}}_i^C=\frac{\sum \limits_{j=1}^{g_1}{x}_{ij}}{g_1};\kern1.25em {\overline{X}}_i^D=\frac{\sum \limits_{j={g}_1+1}^n{x}_{ij}}{n-{g}_1};\kern0.5em {S}_{iC}^2=\frac{1}{g_1-1}\sum \limits_{j=1}^{g_1}{\left({x}_{ij}-{\overline{X}}_i^C\right)}^2\kern0.75em ;\kern0.5em {S}_{iD}^2=\frac{1}{n-{g}_1-1}\sum \limits_{j={g}_1+1}^n{\left({x}_{ij}-{\overline{X}}_i^D\right)}^2\\ {};{S}_i^2=\frac{\left({g}_1-1\right){S}_{iC}^2+\left(n-{g}_1-1\right){S}_{iD}^2}{n-2}.\end{array}} $$

The value from eq. (2) is compared with Student’s *t*-value with *n* − 2 degrees of freedom.

If (*σ*_*iC*_^2^ ≠ *σ*_*iD*_^2^) then the test statistic is

3 $$ t=\frac{{\overline{X}}_i^C-{\overline{X}}_i^D}{\sqrt{\left(\frac{S_{iC}^2}{g_1}+\frac{S_{iD}^2}{n-{g}_1}\right)}} $$

In both cases, the *p*-value is calculated using

4 $$ p-\mathrm{value}=\underset{t_{calculated}}{\overset{\infty }{\int }}f(t) dt $$

**Fig. 1:**
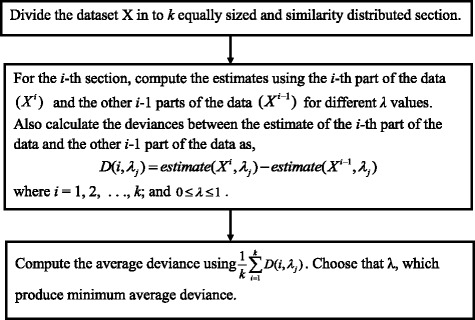
Flowchart of λ selection procedure

In CVP, the *FC* value from (1) and *t*-value from (2) or (3) are calculated using the classical mean and variance. Because the classical mean and variance are influenced by outliers, we propose RVP using the weighted mean and variance instead of the classical mean and variance. For the weighted mean and variance, we use the kernel weight function ${w}_j=\exp \left\{-\frac{\lambda }{2{\left( mad\left({x}_j\right)\right)}^2}{\left({x}_{ij}- median\left({x}_j\right)\right)}^2\right\},$ where *mad* is the median absolute deviation. The value of this weight function lies between zero and one, and is close to zero if the observation is far from the median and close to one if the observation is close to the median. In the weight function, the tuning parameter *λ* is selected using *k*-fold cross validation (Fig. 1 summarizes the *λ* selection procedure). If the dataset does not contain outliers, then the value of *λ* is zero and all the weights are equal to 1, so the method is the same as the classical approach. The steps for RVP are given below.

- **Step − 1**. Calculate *log*_*2*_ (fold change) for the *i*-th metabolite as ${\log}_2\left({FC}_i\right)={\log}_2\left(\frac{{\overline{X}}_i^D}{{\overline{X}}_i^C}\right),$ where ${\overline{X}}_i^D=\sum \limits_{j={g}_1+1}^n{w}_j{x}_{ij}/\left(n-{g}_1\right)$ represents the weighted average intensity of the *i*-th metabolite for the disease group and ${\overline{X}}_i^C=\sum \limits_{j=1}^{g_1}{w}_j{x}_{ij}/{g}_1$ represents the weighted average intensity of the *i*-th metabolite for the control group.
- **Step − 2**. Using the weighted average and weighted variance instead of the classical mean and variance, calculate *-log*_10_(*p*-value) for the *i*-th metabolite from the *t*-test using eqs. (2), (3) and (4), where ${S}_{iC}^2=\sum \limits_{j=1}^{g_1}{w}_j{\left({x}_{ij}-{\overline{X}}_i^C\right)}^2/\left({g}_1-1\right)$ and ${S}_{iD}^2=\sum \limits_{j={g}_1+1}^n{w}_j{\left({x}_{ij}-{\overline{X}}_i^D\right)}^2/\left(n-{g}_1-1\right).$
- **Step − 3**. Draw a scatter plot with log_2_ (fold-change) on the X-axis and *-log*_10_ (*p*-value) from the *t*-test on the Y-axis. This plot is considered to be an outlier-robust volcano plot (RVP). A metabolite is said to be differential if *p*-value < 0.05 and | log_2_ (fold-change) | > 1.

The R package of the proposed method with its user manual is available at https://github.com/nishithkumarpaul/Rvolcano.

Any user can install the “Rvolcano” package in R platform from the GitHub using the following code

library(devtools)

install_github("Rvolcano","nishithkumarpaul")

library(Rvolcano)

To draw the robust volcano plot using the package, the user manual is available at GitHub website.

### Dataset description

In this paper, we use an artificially generated dataset and an experimentally measured metabolomics dataset to evaluate the performance of the proposed method in comparison with nine other methods.

### Artificial data

In this study, as in [6], we generate an artificial metabolomics dataset using a one-way ANOVA model *y*_*ijk*_ = *μ*_*i*_ + *g*_*ij*_ + ∈_*ijk*_, where *y*_*ijk*_ is the intensity of the *i*^th^ metabolite, *j*^th^ group and *k*^th^ sample, *μ*_*i*_ denotes the overall intensity of metabolite *i*, *g*_*ij*_ is the *j*^th^ group effect for the *i*^th^ metabolite, and ∈_*ijk*_ is a random error term. In this linear model, *μ*_*i*_ ~ *uniform* (10, 20) and ∈_*ijk*_ ~ N(0,1). The disease and control group effects for increased concentrations of metabolites are *g*_*ij*_ = *N*(4, 1) and *g*_*ij*_ = *N*(2, 1), respectively; for decreased concentrations of metabolites, we use *g*_*ij*_ = *N*(2, 1) and *g*_*ij*_ = *N*(4, 1) for the disease and control groups, respectively. Both group effects for non-differential (equal concentration) metabolites are *g*_*ij*_ = *N*(0, 1). To create the artificial metabolomics dataset, we designated 130 metabolites as non-differential and 20 metabolites as differential (having differential concentrations). The dataset contained 70 subjects with 40 subjects in group-1 and 30 subjects in group-2. To investigate the performance of the proposed method under different conditions, outliers were randomly distributed in the artificially generated data matrix at different rates (5%, 10%, 15%, 20%, and 25%). Note that these outliers can fall anywhere in the data matrix. The outliers for the *i*-th metabolite were taken from a normal distribution with mean 3*μ_*i*_ and variance ${\sigma}_i^2$, i.e. N (3*μ_*i*_, ${\sigma}_i^2$), where μ_i_ and ${\sigma}_i^2$ are the mean and variance of the *i*-th metabolite. In total, 500 artificial datasets were generated for each condition, and the performance of the proposed method was evaluated using these datasets.

### Experimentally measured data

In this paper, we considered a well-known publicly available metabolomics dataset for breast cancer serum data and control serum data containing metabolite abundance level measurements from different subjects. This dataset is available from the National Institute of Health (NIH) data repository and was collected by the University of Hawaii Cancer Center under study ID ST000356. The data were measured using a gas chromatography with time-of-flight mass spectrometry (GC-TOFMS) instrument and quantified using the ChromaTOF software (v4.33, Leco Co, CA, USA). The dataset contains 134 subjects (103 breast cancer without any treatment and 31 control subjects) and 101 metabolites. Auto-scaling was used to reduce the systematic variation in the dataset. To investigate the performance of the proposed method under different conditions, we also modified the dataset by changing 5%, 10%, and 15% of the real values by *N*(4 × *μ*_*i*_, *σ*_*i*_^*2*^), where *μ*_*i*_ and *σ*_*i*_^*2*^ are the mean and variance of the *i-*th metabolite in the breast cancer data matrix.

## Results and discussion

The performance of our proposed method was compared with nine existing methods using both the artificial and experimental datasets.

### Performance evaluation based on artificially generated data

**Fig. 2:**
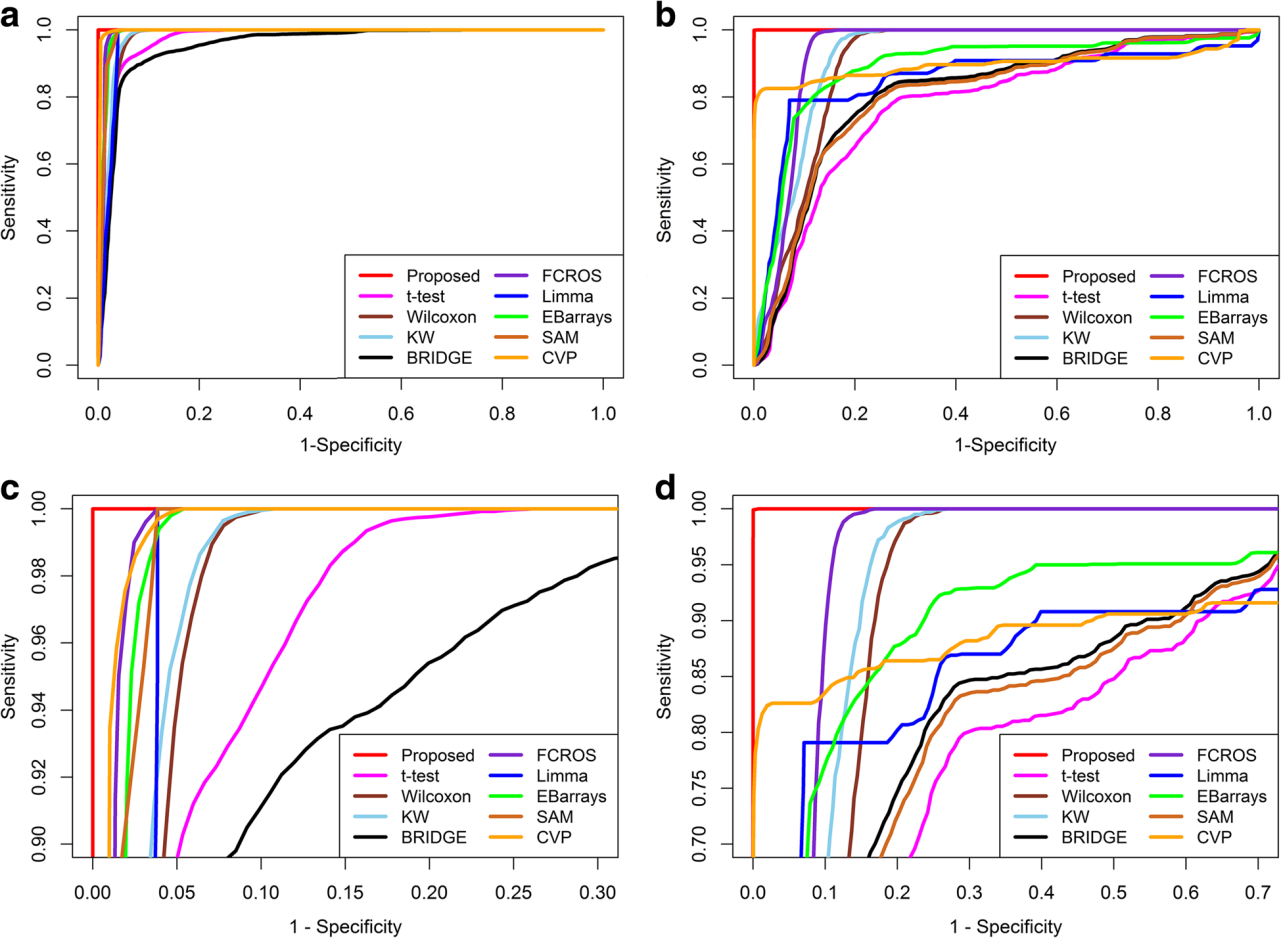
Performance evaluation using ROC curves for different differential metabolite identification techniques **a** in the absence of outliers, **b** with 15% outliers, **c** zoom image of upper left region in absence of outliers, and **d** zoom image of upper left region with 15% outliers

**Fig. 3:**
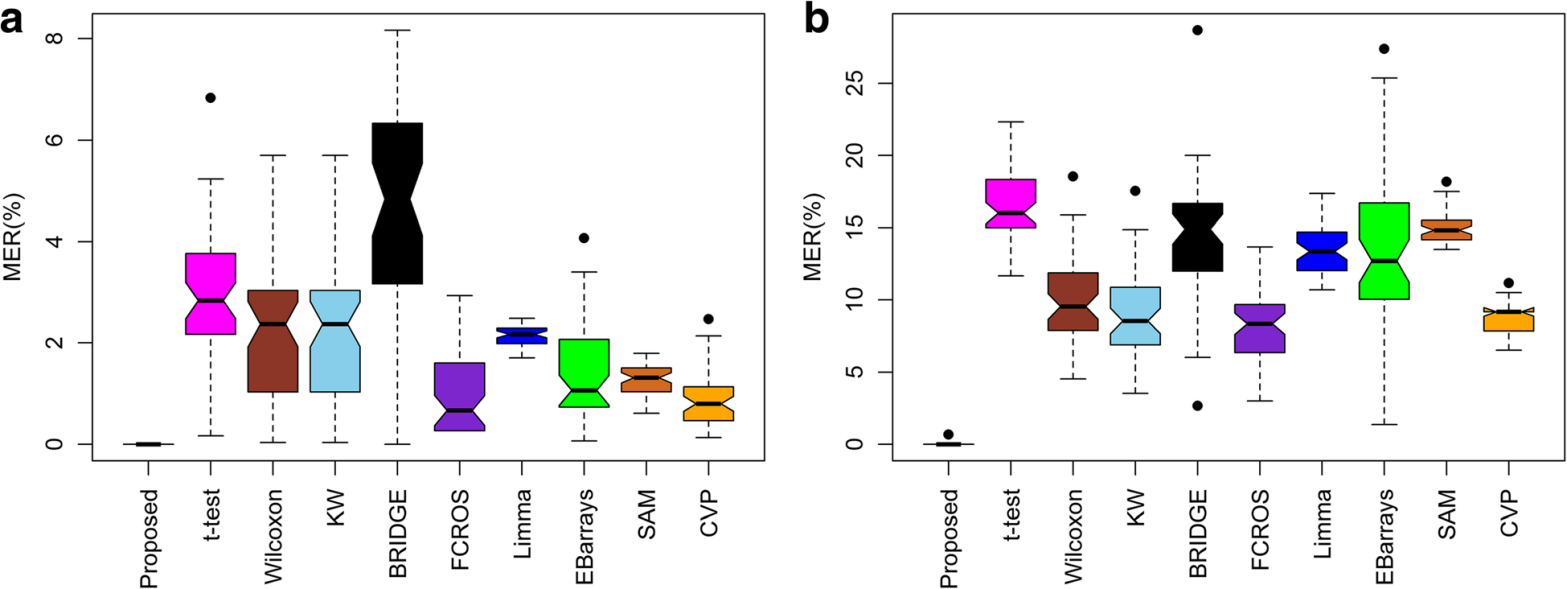
Performance evaluation using box plots of 500 misclassification error rates (MERs) for different differential metabolite identification techniques **a** in the absence of outliers, and **b** with 15% outliers

**Fig. 4:**
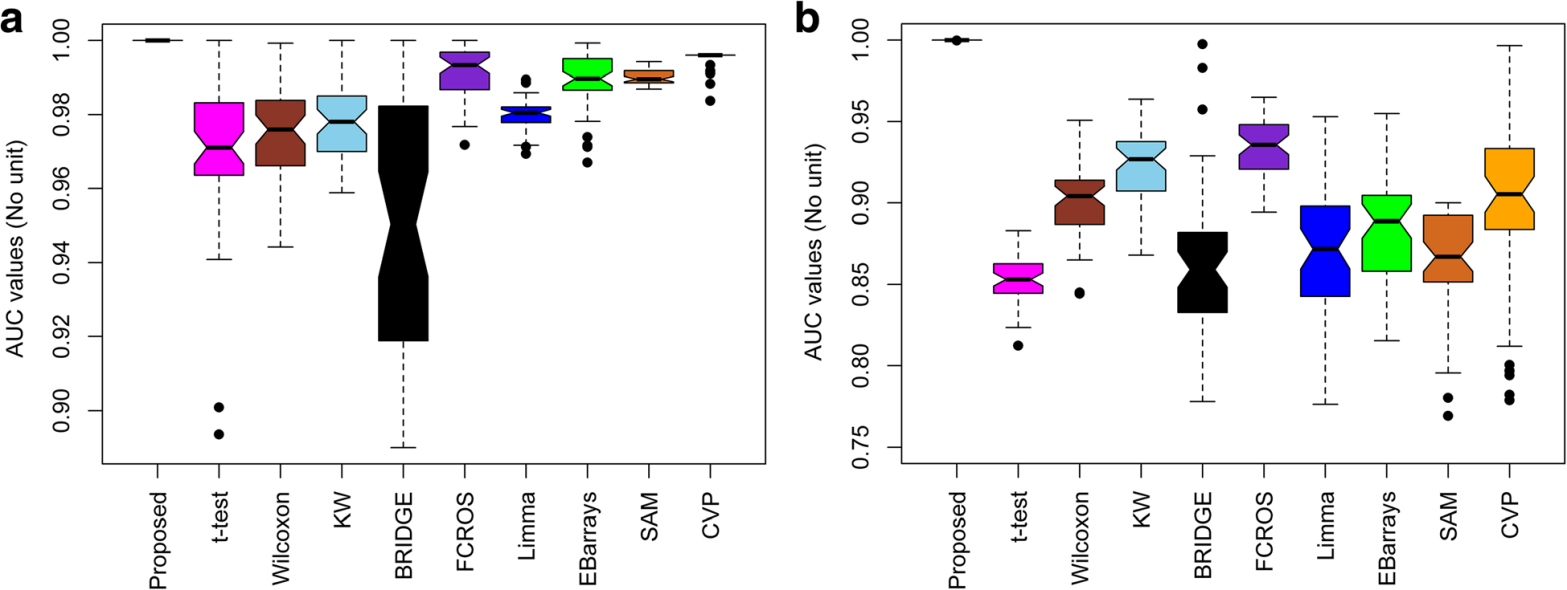
Performance evaluation using box plots of 500 AUC values for different differential metabolite identification techniques **a** in the absence of outliers, and **b** with 15% outliers

The performance of the proposed method was compared with those of nine existing methods using 500 artificial datasets. The misclassification error rates (MERs) for differential metabolite identification were calculated for each method. The true positive rate (TPR), false positive rate (FPR), true negative rate (TNR), false negative rate (FNR), the area under the receiver operating characteristic (ROC) curve (AUC), and the partial AUC (pAUC with *FPR* ≤ 0.2) were also calculated. The above performance indices were calculated both in the absence and presence of outliers. The average MER, AUC and pAUC values for the artificial datasets are shown in the Additional file 1: Table S1. A method with a lower MER value and higher AUC and pAUC values is considered better. From Additional file 1: Table S1, we observe that our proposed method gave a lower MER value and higher AUC and pAUC values both in the absence and presence of outliers. We also present the ROC curves in Fig. 2 and boxplots of the 500 MER and AUC values in Figs. 3 and 4, respectively. Figure 2 shows that our proposed method gave a higher average TPR with respect to average FPR in comparison with the existing methods both in the absence of outliers and with 15% outliers. In Fig. 3, it is clear that the proposed method produced a smaller MER with minimum variability, and in Fig. 4, the proposed method gave higher AUC values with minimum variability both in the absence of outliers and with 15% outliers. To check the robustness of the different methods, we plotted the ROC curve and a boxplot of MER and AUC values for the artificial datasets for different rates (0%, 5%, 10%, 15%, 20% and 25%) of outliers. The graphs are shown in Additional file 2: Figures S1, S2 and S3. Further more, to measure the efficiency of the proposed method, we also calculated the power and false discovery rate (FDR) for small sample in both absence and presence of outliers (Additional file 1: Table S2). In Additional file 1: Table S2, the proposed method gave higher power and lower FDR in absence and presence of outliers for small sample sizes. More over, we calculated the execution time (speed of execution) in seconds of different methods including the proposed one for different number of metabolites and samples (Additional file 1: Table S3). Additional file 1: Table S3 showed that it is seen that the execution time of the proposed technique was lower than the robust Bayesian technique BRIDGE in all the cases, but this time is comperatively higher than the execution time of other techniques. This is one of the limitations of the proposed technique. Another limitation of the proposed technique is its compatibility only for the analyses between two groups. Although the proposed technique has several limitations, on the basis of above analyses of artificial datasets, we could conclude that the proposed RVP-based differential metabolite identification technique performs better than the nine existing methods.

### Performance evaluation based on experimentally measured data

**Fig. 5:**
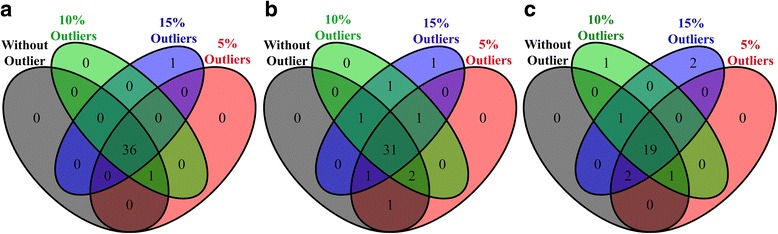
Performance evaluation using Venn diagrams for the number of differential metabolites identified by **a** RVP, **b** Limma, and **c** FCROS for the experimental dataset

For the experimentally measured metabolomics (breast cancer) data, the performance of the proposed method was measured using differential metabolite identification from the experimental dataset and the modified experimental datasets. The experimental data was modified by artificially incorporating different rates (5%, 10% and 15%) of outliers. Methods that identified similar combinations of differential metabolites for the experimental dataset and the modified datasets were considered to be more outlier-robust. Additional file 1: Table S4 shows the number of differential metabolites identified by different methods for the original and modified datasets. While Additional file 1: Table S4 shows any differences in the number of differential metabolites identified by each method for the datasets in the absence and presence of outliers, we also need to know which methods identified similar combinations of differential metabolites. We use Venn diagrams to find methods that gave similar combinations of differential metabolites in the absence and presence of outliers. From Additional file 1: Table S4, we chose three techniques (Limma, FCROS and the proposed method) according to the lowest variability in the number of differential metabolites. The corresponding Venn diagrams are presented in Fig. 5. Venn diagrams for all methods are given in the Additional file 2: Figure S4. From Fig. 5, we observe that our proposed method produced similar combinations of differential metabolites in the absence and presence of outliers. Therefore, we conclude that our proposed method performs better than the nine existing techniques.

**Fig. 6:**
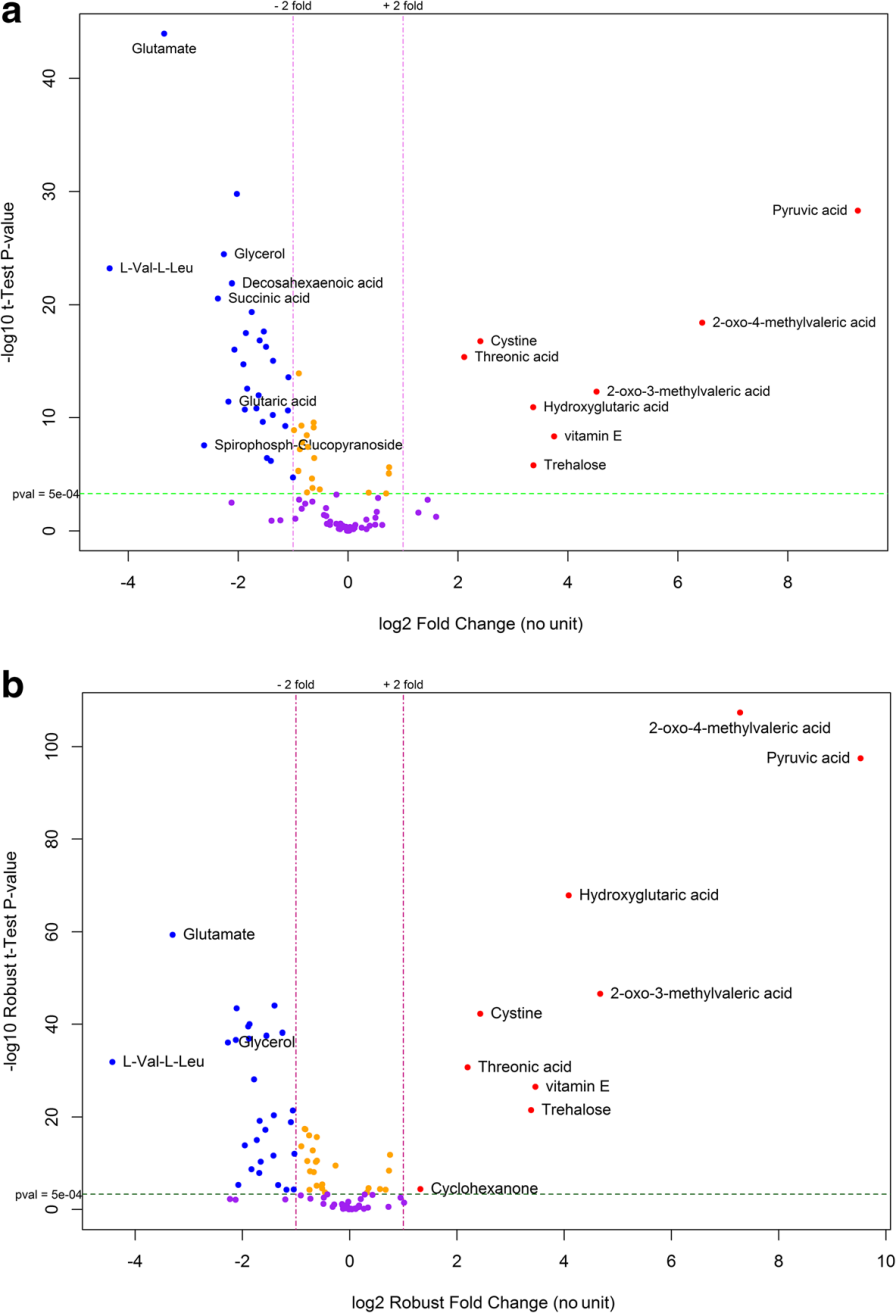
Differential metabolite identification for the experimental dataset using **a** CVP, and **b** RVP

Because we modified CVP to create an outlier-robust version called RVP, we also examine the results of these two methods for the experimental data. For the experimental dataset, CVP identified 36 metabolites as differential, whereas our proposed RVP identified 37 metabolites as differential (Fig. 6). The same 36 metabolites were identified by both methods, while RVP also identified cyclohexanone. From reviewing the literature, we found that cyclohexanone is a metabolite that is associated with breast cancer as well as several other cancer diseases (Table 1) [35–40]. This suggests that our method is more reliable for differential metabolite identification.

**Table 1:** Literature review of cyclohexanone metabolite associated diseases

Authors	Disease	Title of the paper	Journal name
Westhoff et al., 2010 [35]	Lung cancer	Differentiation of chronic obstructive pulmonary disease (COPD) including lung cancer from healthy control group by breath analysis using ion mobility spectrometry	International Journal for Ion Mobility Spectrometry
Wei et al., 2012 [36]	Prostate cancer	Effects of cyclohexanone analogues of curcumin on growth, apoptosis and NF-κB activity in PC-3 human prostate cancer cells	Oncology letters
Leung et al. 2012 [37]	Breast cancer	Identification of cyclohexanone derivatives that act as catalytic inhibitors of topoisomerase I: effects on tamoxifen-resistant MCF-7 cancer cells	Investigational new drugs
Wang et al., 2014 [26]	Breast cancer	Volatile Organic Metabolites Identify Patients with Breast Cancer, Cyclomastopathy, and Mammary Gland Fibroma	Scientific Report (Nature)
Mochalski et al., 2014 [38]	Renal disease	Blood and breath profiles of volatile organic compounds in patients with end-stage renal disease	BMC Nephrology
Liu et al., 2014 [39]	Lung cancer	Investigation of volatile organic metabolites in lung cancer pleural effusions by solid-phase microextraction and gas chromatography/mass spectrometry	Journal of Chromatography
Silva et al., 2017 [40]	Breast cancer	Volatile metabolomic signature of human breast cancer cell lines	Scientific Report (Nature)

**Fig. 7:**
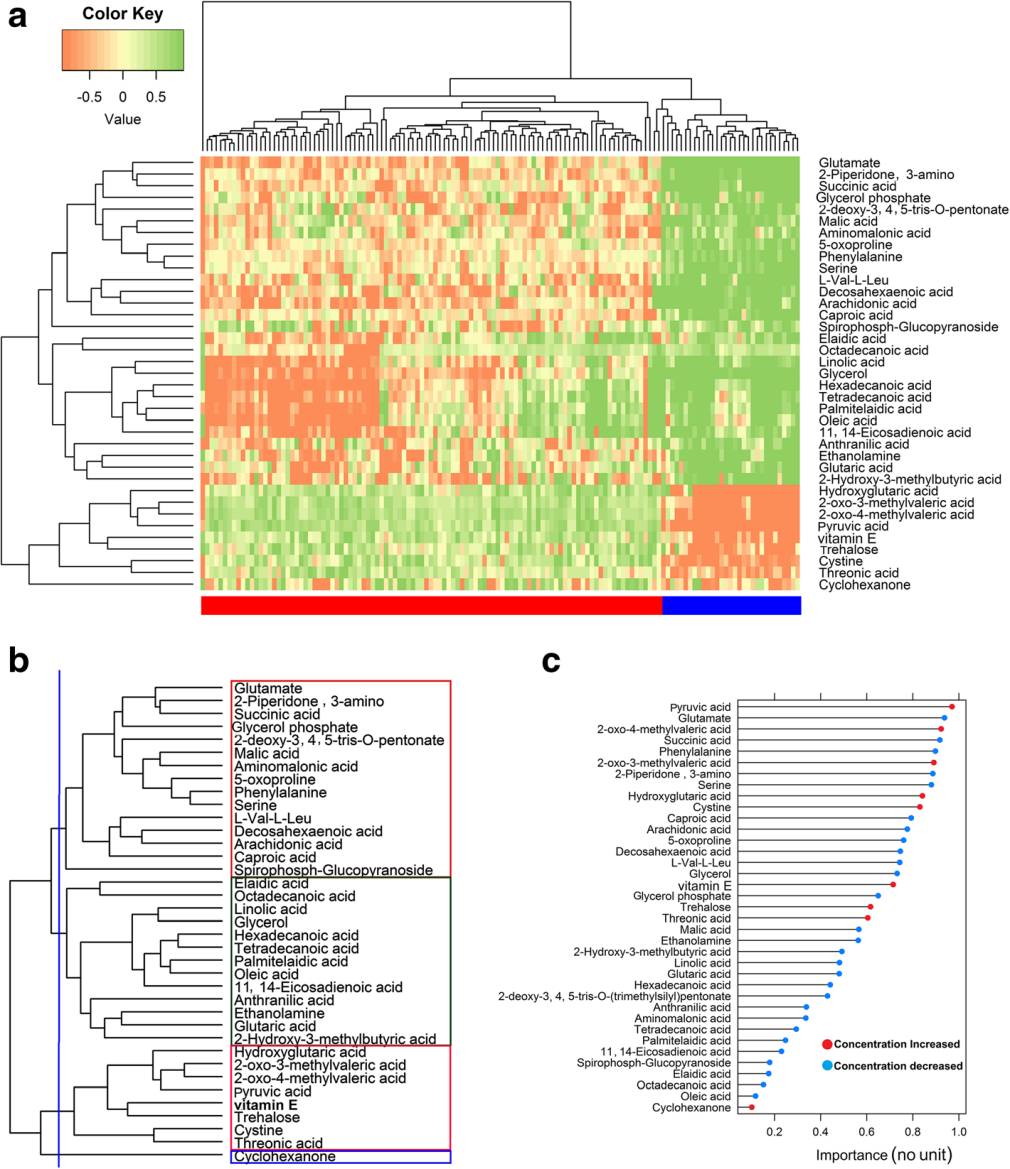
Metabolomic biomarker identification for breast cancer. **a** Heatmap plot of up-regulation and down-regulation for the 37 differential metabolites identified by the proposed method (red indicates cancer samples and blue indicates control samples). **b** Clustering of the 37 differential metabolites for the experimental dataset. Hierarchical clustering was used after normalizing the experimentally measured breast cancer dataset by auto-scaling. **c** Ranking of the 37 differential metabolites according to the importance score calculated using an SVM classifier with radial basis kernel function

Sometimes, a set of metabolites may show the same pattern of behavior, in that if one of them is differential then the whole set is identified as differential. To identify the potential biomarkers from the 37 differential metabolites identified by RVP, we clustered the differential metabolites using hierarchical clustering (Fig. 7) and found the most important metabolites in each cluster (the importance score is calculated using a support vector machine (SVM) classifier with radial basis kernel function) (Fig. 7). From Fig. 7 (b), we obtained four clusters and chose the most important metabolite from each cluster according to the importance score in Fig. 7 (c). For the first cluster in Fig. 7 (b), there are 15 metabolites of which the most important is glutamate. Similarly, ethanolamine is the most important metabolite for the second cluster, pyruvic acid for the third cluster and cyclohexanone for the fourth cluster. These four metabolites (glutamate, ethanolamine, pyruvic acid, and cyclohexanone) may thus be biomarkers for breast cancer. Laboratory-based targeted metabolomics analysis to test this hypothesis could be an avenue for future research.

## Conclusions

Outlying observations weaken the performance of existing differential metabolite identification techniques. In this paper, we have proposed a new outlier-robust differential metabolite identification technique for identifying differential metabolites in the presence of outliers. To investigate the performance of our proposed method, we analyzed artificial data and experimental data in the absence and presence of outliers. We also compared the performance of our proposed method with nine existing differential metabolite identification techniques using the ROC curve, and the average MER, AUC and pAUC values. Both the artificial and experimental data analysis show that our proposed method performed better. The proposed RVP also identified an additional metabolite (cyclohexanone) that was overlooked by CVP, and it has been shown that this metabolite is associated with several cancer diseases. We recommend using the proposed method to identify differential metabolites from noisy metabolomics datasets.

## Additional files

Additional file 1: **Table S1.** Performance evaluations for different methods using average MER, AUC and pAUC values. **Table S2.** Efficiency Calculation of different techniques using power and FDR in both absence and presence of outliers for small sample sizes. For this analysis 1500 metabolites have been taken in the dataset. **Table S3.** Execution time calculation in seconds of different methods including the proposed one for different number of metabolites and different number of samples (Computer Configuration: Processor-Intel Core i7 3.6 GHz, RAM-16.0GB, OS- 64 bit & Windows 8). **Table S4.** Number of differential metabolites identified by different methods. (DOC 136 kb)

Additional file 2: Performance evaluation of the proposed technique compared to other techniques using ROC curves and MER and AUC values for the artificial datasets in the absence and presence of outliers. **Figure S1.** Performance evaluation using ROC curves for different differential metabolite identification techniques (a) in the absence of outliers, (b) with 5% outliers, (c) with 10% outliers, (d) with 15% outliers, (e) with 20% outliers, and (f) with 25% outliers. **Figure S2.** Performance evaluation using box plots of 500 MERs for different differential metabolite identification techniques (a) in the absence of outliers, (b) with 5% outliers, (c) with 10% outliers, (d) with 15% outliers, (e) with 20% outliers, and (f) with 25% outliers. **Figure S3.** Performance evaluation using box plots of 500 AUC values for different differential metabolite identification techniques (a) in the absence of outliers, (b) with 5% outliers, (c) with 10% outliers, (d) with 15% outliers, (e) with 20% outliers, and (f) 25% outliers. **Figure S4.** Performance evaluation using Venn diagrams for the number of differential metabolites identified by different differential metabolite identification methods for the experimental dataset. (DOC 6677 kb)

## Abbreviations

ANOVA: Analysis of variance
AUC: Area under the ROC curve
BRIDGE: Bayesian robust inference for differential gene expression
CVP: Classical volcano plot
FC: Fold change
FCROS: Fold change rank ordering statistics
FNR: False negative rate
FPR: False positive rates
GC-MS: Gas chromatography mass spectrometry
GC-TOFMS: Gas chromatography with time-of-flight mass spectrometry
KW: Kruskal–Wallis
LC-MS: Liquid chromatography mass spectrometry
Limma: Linear models for microarray
MER: Misclassification error rate
NIH: National institute of health
pAUC: Partial area under the ROC curve
ROC: Receiver operating characteristic
RVP: Robust volcano plot
SAM: Significant analysis of microarray
SVM: Support vector machine
TNR: True negative rate
TPR: True positive rates

## References

[1] Gieger C, Geistlinger L, Altmaier E, De Angelis MH, Kronenberg F, Meitinger T, Mewes HW, Wichmann HE, Weinberger KM, Adamski J, Illig T (2008). Genetics meets metabolomics: a genome-wide association study of metabolite profiles in human serum. PLoS Genet.

[2] Fiehn O (2002). Metabolomics—the link between genotypes and phenotypes. Functional Genomics.

[3] Newgard CB (2017). Metabolomics and metabolic diseases: where do we stand?. Cell Metab.

[4] Wang TJ, Larson MG, Vasan RS, Cheng S, Rhee EP, McCabe E, Lewis GD, Fox CS, Jacques PF, Fernandez C, O'donnell CJ (2011). Metabolite profiles and the risk of developing diabetes. Nat Med.

[5] Sumner LW, Mendes P, Dixon RA (2003). Plant metabolomics: large-scale phytochemistry in the functional genomics era. Phytochemistry.

[6] Zhan X, Patterson AD, Ghosh D (2015). Kernel approaches for differential expression analysis of mass spectrometry-based metabolomics data. BMC Bioinformatics.

[7] Mamas M, Dunn WB, Neyses L, Goodacre R (2011). The role of metabolites and metabolomics in clinically applicable biomarkers of disease. Arch Toxicol.

[8] Trusheim MR, Berndt ER, Douglas FL (2007). Stratified medicine: strategic and economic implications of combining drugs and clinical biomarkers. Nat Rev Drug Discov.

[9] Karpievitch YV, Dabney AR, Smith RD (2012). Normalization and missing value imputation for label-free LC-MS analysis. BMC Bioinformatics.

[10] Hrydziuszko O, Viant MR (2012). Missing values in mass spectrometry based metabolomics: an undervalued step in the data processing pipeline. Metabolomics.

[11] Armitage EG, Godzien J, Alonso-Herranz V, López-Gonzálvez Á, Barbas C (2015). Missing value imputation strategies for metabolomics data. Electrophoresis.

[12] Gromski PS, Xu Y, Kotze HL, Correa E, Ellis DI, Armitage EG, Turner ML, Goodacre R (2014). Influence of missing values substitutes on multivariate analysis of metabolomics data. Meta.

[13] Yang J, Zhao X, Lu X, Lin X, Xu G (2015). A data preprocessing strategy for metabolomics to reduce the mask effect in data analysis. Front Mol Biosci.

[14] Steuer R, Morgenthal K, Weckwerth W, Selbig J (2007). A gentle guide to the analysis of metabolomic data. Metabolomics: Methods and Protocols.

[15] DeHaven CD, Evans AM, Dai H, Lawton KA (2010). Organization of GC/MS and LC/MS metabolomics data into chemical libraries. J Cheminform.

[16] Godzien J, Ciborowski M, Angulo S, Barbas C (2013). From numbers to a biological sense: How the strategy chosen for metabolomics data treatment may affect final results. A practical example based on urine fingerprints obtained by LC-MS. Electrophoresis.

[17] Blanchet L, Smolinska A (2016). Data fusion in metabolomics and proteomics for biomarker discovery. Statistical Analysis in Proteomics.

[18] Kumar N, Hoque MA, Shahjaman M, Islam SMS, Mollah MNH. Metabolomic biomarker identification in presence of outliers and missing values. Biomed Res Int. 2017;2017:1–11. 10.1155/2017/2437608 PMC5331169 28293630

[19] Snyder MN, Henderson WM, Glinski DA, Purucker ST (2017). Biomarker analysis of American toad (Anaxyrus americanus) and grey tree frog (Hyla versicolor) tadpoles following exposure to atrazine. Aquat Toxicol.

[20] Bordbar A, Yurkovich JT, Paglia G, Rolfsson O, Sigurjónsson ÓE, Palsson BO (2017). Elucidating dynamic metabolic physiology through network integration of quantitative time-course metabolomics. Sci Rep.

[21] Fan Y, Zhou X, Xia TS, Chen Z, Li J, Liu Q, Alolga RN, Chen Y, Lai MD, Li P, Zhu W (2016). Human plasma metabolomics for identifying differential metabolites and predicting molecular subtypes of breast cancer. Oncotarget.

[22] Li W (2012). Volcano plots in analyzing differential expressions with mRNA microarrays. J Bioinforma Comput Biol.

[23] Dembélé D, Kastner P (2014). Fold change rank ordering statistics: a new method for detecting differentially expressed genes. BMC Bioinformatics.

[24] Tusher VG, Tibshirani R, Chu G (2001). Significance analysis of microarrays applied to the ionizing radiation response. Proc Natl Acad Sci.

[25] McMillan A, Renaud JB, Gloor GB, Reid G, Sumarah MW (2016). Post-acquisition filtering of salt cluster artefacts for LC-MS based human metabolomic studies. J Cheminform.

[26] Wang C, Sun B, Guo L, Wang X, Ke C, Liu S, Zhao W, Luo S, Guo Z, Zhang Y, Xu G (2014). Volatile organic metabolites identify patients with breast cancer, cyclomastopathy, and mammary gland fibroma. Sci Rep.

[27] Gottardo R, Raftery AE, Yee Yeung KA, Bumgarner RE (2006). Bayesian robust inference for differential gene expression in microarrays with multiple samples. Biometrics.

[28] Kendziorski CM, Newton MA, Lan H, Gould M (2003). On parametric empirical Bayes methods for comparing multiple groups using replicated gene expression profiles. Stat Med.

[29] Smyth GK (2005). Limma: linear models for microarray data. Bioinformatics and computational biology solutions using R and Bioconductor.

[30] Efron B, Tibshirani R, Storey JD, Tusher V (2001). Empirical Bayes analysis of a microarray experiment. J Am Stat Assoc.

[31] Do KA, Müller P, Tang F (2005). A Bayesian mixture model for differential gene expression. J R Stat Soc: Ser C: Appl Stat.

[32] Mollah MM, Mollah MN, Kishino H (2012). β-empirical Bayes inference and model diagnosis of microarray data. BMC Bioinformatics.

[33] Jung K, Friede T, Beißbarth T (2011). Reporting FDR analogous confidence intervals for the log fold change of differentially expressed genes. BMC Bioinformatics.

[34] Zhang S, Cao J (2009). A close examination of double filtering with fold change and t test in microarray analysis. BMC Bioinformatics.

[35] Westhoff M, Litterst P, Maddula S, Bödeker B, Rahmann S, Davies AN, Baumbach JI (2010). Differentiation of chronic obstructive pulmonary disease (COPD) including lung cancer from healthy control group by breath analysis using ion mobility spectrometry. Int J Ion Mobil Spectrom.

[36] Wei X, Du ZY, Cui XX, Verano M, Mo RQ, Tang ZK, Conney AH, Zheng X, Zhang K (2012). Effects of cyclohexanone analogues of curcumin on growth, apoptosis and NF-κB activity in PC-3 human prostate cancer cells. Oncol Lett.

[37] Leung E, Rewcastle GW, Joseph WR, Rosengren RJ, Larsen L, Baguley BC (2012). Identification of cyclohexanone derivatives that act as catalytic inhibitors of topoisomerase I: effects on tamoxifen-resistant MCF-7 cancer cells. Investig New Drugs.

[38] Mochalski P, King J, Haas M, Unterkofler K, Amann A, Mayer G (2014). Blood and breath profiles of volatile organic compounds in patients with end-stage renal disease. BMC Nephrol.

[39] Liu H, Wang H, Li C, Wang L, Pan Z, Wang L (2014). Investigation of volatile organic metabolites in lung cancer pleural effusions by solid-phase microextraction and gas chromatography/mass spectrometry. J Chromatogr B.

[40] Silva CL, Perestrelo R, Silva P, Tomás H, Câmara JS (2017). Volatile metabolomic signature of human breast cancer cell lines. Sci Rep.

