# Supplementary material for: Robust volcano plot: identification of differential metabolites in the presence of outliers
Source: BMC Bioinformatics. 2018 Apr 11;19:128. doi: 10.1186/s12859-018-2117-2 (PMC5896081; doi:10.1186/s12859-018-2117-2)
Supplement: Supplementary file 1 — Table S1. Performance evaluations for different methods using average MER, AUC and pAUC values. Table S2. Efficiency Calculation of different techniques using power and FDR in both absence and presence of outliers for small sample sizes. For this analysis 1500 metabolites have been taken in the dataset. Table S3. Execution time calculation in seconds of different methods including the proposed one for different number of metabolites and different number of samples (Computer Configuration: Processor-Intel Core i7 3.6 GHz, RAM-16.0GB, OS- 64 bit & Windows 8). Table S4. Number of differential metabolites identified by different methods. (DOC 136 kb) [file 12859_2018_2117_MOESM1_ESM.doc]

# Additional file 1:

**Table S1.** Performance evaluations for different methods using average MER, AUC and pAUC values.

| **Identification**  **Techniques** | **Different Measures** | **Without**  **Outliers** | **5% Outliers** | **10% Outliers** | **15% Outliers** | **20% Outliers** | **25% Outliers** |
| --- | --- | --- | --- | --- | --- | --- | --- |
| **Proposed** | MER | **0** | **0** | **0** | **0.0133** | **0.1600** | **0.7733** |
|  | AUC | **1** | **1** | **1** | **0.9999** | **0.9991** | **0.9950** |
|  | pAUC | **0.2** | **0.2** | **0.2** | **0.1999** | **0.1991** | **0.1958** |
| ***t*-test** | MER | 2.897 | 8.125 | 11.59 | 16.35 | 29.85 | 34.65 |
|  | AUC | 0.9711 | 0.9187 | 0.8821 | 0.8519 | 0.6929 | 0.6588 |
|  | pAUC | 0.1711 | 0.1187 | 0.0915 | 0.0722 | 0.0431 | 0.0415 |
| **Wilcoxon** | MER | 2.267 | 5.544 | 6.153 | 10.01 | 12.32 | 16.75 |
|  | AUC | 0.9752 | 0.9428 | 0.9488 | 0.9007 | 0.8806 | 0.8419 |
|  | pAUC | 0.1752 | 0.1523 | 0.1491 | 0.1016 | 0.0841 | 0.0793 |
| **KW** | MER | 2.267 | 5.544 | 6.091 | 9.012 | 10.17 | 15.67 |
|  | AUC | 0.9781 | 0.9428 | 0.9491 | 0.9242 | 0.9091 | 0.8501 |
|  | pAUC | 0.1781 | 0.1523 | 0.1497 | 0.1207 | 0.1109 | 0.0828 |
| **BRIDGE** | MER | 4. 5933 | 8.9991 | 10.7823 | 15.2657 | 26.1200 | 26.0067 |
|  | AUC | 0.9544 | 0.9023 | 0.8893 | 0.8573 | 0.7555 | 0.7537 |
|  | pAUC | 0.9432 | 0.1418 | 0.1107 | 0.0939 | 0.0577 | 0.0573 |
| **FCROS** | MER | 0.8800 | 2.875 | 6.293 | 8.907 | 9.653 | 13.59 |
|  | AUC | 0.9911 | 0.9647 | 0.9483 | 0.9341 | 0.9167 | 0.8708 |
|  | pAUC | 0.1911 | 0.1647 | 0.1483 | 0.1341 | 0.1272 | 0.0908 |
| **Limma** | MER | 2.133 | 2.932 | 7.684 | 13.64 | 18.19 | 19.99 |
|  | AUC | 0.9798 | 0.9682 | 0.9176 | 0.8702 | 0.8257 | 0.8006 |
|  | pAUC | 0.1798 | 0.1702 | 0.1217 | 0.1207 | 0.1158 | 0.1068 |
| **Ebarrays** | MER | 1.367 | 8.093 | 8.215 | 13.14 | 28.96 | 31.95 |
|  | AUC | 0.9894 | 0.9205 | 0.9091 | 0.8776 | 0.7017 | 0.6924 |
|  | pAUC | 0.1894 | 0.1205 | 0.1253 | 0.1246 | 0.0434 | 0.0536 |
| **SAM** | MER | 1.167 | 2.793 | 9.792 | 15.27 | 27.87 | 32.01 |
|  | AUC | 0.9901 | 0.9663 | 0.8989 | 0.8567 | 0.7146 | 0.6844 |
|  | pAUC | 0.1901 | 0.1725 | 0.1117 | 0.0936 | 0.0440 | 0.0516 |
| **CVP** | MER | 0.7933 | 1.433 | 3.073 | 9.667 | 20.45 | 32.02 |
|  | AUC | 0.9951 | 0.9867 | 0.9701 | 0.9067 | 0.8011 | 0.6840 |
|  | pAUC | 0.1951 | 0.1886 | 0.1723 | 0.1659 | 0.1373 | 0.0515 |

Note: The average MER, AUC and pAUC values were calculated using 500 artificial datasets both in the absence and presence of (artificially imputed) outliers.

**Table S2:** Efficiency Calculation of different techniques using power and FDR in both absence and presence of outliers for small sample sizes. For this analysis 1500 metabolites have been taken in the dataset.

| **Identification**  **Techniques** | **Performance Indices** | **20 samples(10 samples in each group)** | | **10 samples (5 samples in each group)** | |
| --- | --- | --- | --- | --- | --- |
|  |  | **Without Outliers** | **5% Outliers** | **Without Outliers** | **5% Outliers** |
| Proposed | Power | 1 | 0.995 | 1 | 1 |
|  | FDR | 0 | 0 | 0 | 0.005 |
| KW | Power | 1 | 0.985 | 1 | 0.775 |
|  | FDR | 0.024 | 0.294 | 0.283 | 0.337 |
| BRIDGE | Power | 1 | 0.950 | 1 | 0.865 |
|  | FDR | 0 | 0 | 0 | 0 |
| t-test | Power | 1 | 0.750 | 0.975 | 0.678 |
|  | FDR | 0 | 0.065 | 0.045 | 0.224 |
| Wilcox | Power | 1 | 0.985 | 1 | 0.775 |
|  | FDR | 0.087 | 0.139 | 0.122 | 0.165 |
| FCROS | Power | 1 | 0.995 | 1 | 1 |
|  | FDR | 0.285 | 0.344 | 0. 348 | 0.487 |
| Limma | Power | 1 | 0.880 | 1 | 0.680 |
|  | FDR | 0 | 0.140 | 0 | 0.320 |
| EBarrys | Power | 1 | 0.955 | 1 | 0.790 |
|  | FDR | 0.069 | 0.441 | 0.122 | 0.365 |
| SAM | Power | 1 | 0.790 | 0.950 | 0.615 |
|  | FDR | 0 | 0.015 | 0.138 | 0.010 |
| CVP | Power | 1 | 0.920 | 1 | 0.780 |
|  | FDR | 0.005 | 0.128 | 0.224 | 0.187 |

**Table S3:** Execution time calculation in seconds of different methods including the proposed one for different number of metabolites and different number of samples (Computer Configuration: Processor-Intel Core i7 3.6 GHz, RAM-16.0GB, OS- 64 bit & Windows 8).

| **No. of Metabolites** | **No. of Subjects** | **Different Methods** | | | | | | | | | |
| --- | --- | --- | --- | --- | --- | --- | --- | --- | --- | --- | --- |
|  |  | **Proposed** | ***t*-test** | **Wilcoxon** | **KW** | **BRIDGE** | **FCROS** | **Limma** | **Ebarrays** | **SAM** | **CVP** |
| **2500** | **100** | 40.15 | 0.9631 | 1.352 | 2.709 | 63.61 | 2.091 | 1.573 | 0.9893 | 1.005 | 1.331 |
|  | **70** | 21.436 | 0.9063 | 1.008 | 2.224 | 44.81 | 1.161 | 1. 235 | 0.9246 | 0.9452 | 1.124 |
|  | **50** | 15.81 | 0.8649 | 0.8723 | 2.037 | 34.18 | 0.9833 | 1.156 | 0.8764 | 0.8973 | 0.9571 |
|  | **30** | 8.630 | 0.7526 | 0.7779 | 1.518 | 21.62 | 0.6213 | 0.9625 | 0.7531 | 0.7544 | 0.7835 |
| **2000** | **100** | 32.66 | 0.9567 | 1.299 | 2.473 | 54.27 | 1.975 | 1.215 | 0.9601 | 0.9719 | 1.235 |
|  | **70** | 17.24 | 0.8264 | 0.9143 | 1.739 | 36.19 | 1.012 | 1.104 | 0.8528 | 0.9171 | 0.9967 |
|  | **50** | 11.83 | 0.7974 | 0.8529 | 1.451 | 26.75 | 0.9657 | 1.076 | 0.8036 | 0.8621 | 0.9364 |
|  | **30** | 7.031 | 0.5962 | 0.6218 | 1.183 | 17.23 | 0.5594 | 0.9107 | 0.6122 | 0.6729 | 0.6809 |
| **1500** | **100** | 27.74 | 0.8425 | 0.9578 | 1.488 | 39.28 | 1.295 | 0.9898 | 0.8565 | 0.8929 | 0.9527 |
|  | **70** | 17.56 | 0.8296 | 0.8767 | 1.236 | 28.07 | 0.9072 | 0.9381 | 0.8412 | 0.8761 | 0.9072 |
|  | **50** | 9.422 | 0.7603 | 0.8029 | 1.061 | 20.66 | 0.8564 | 0.9076 | 0.7753 | 0.7986 | 0.8753 |
|  | **30** | 5.316 | 0.5082 | 0.5219 | 0.8771 | 13.72 | 0.5062 | 0.8725 | 0.5319 | 0.6128 | 0.6384 |
| **1000** | **100** | 16.31 | 0.5623 | 0.7925 | 0.9810 | 25.07 | 0.9975 | 0.8164 | 0.5764 | 0.6012 | 0.6825 |
|  | **70** | 8.576 | 0.3252 | 0.3954 | 0.8312 | 17.82 | 0.5953 | 0.5834 | 0.3385 | 0.3773 | 0.4128 |
|  | **50** | 5.947 | 0.2716 | 0.3248 | 0.7555 | 13.297 | 0.4657 | 0.5849 | 0.2799 | 0.2914 | 0.3214 |
|  | **30** | 3.461 | 0.1736 | 0.1815 | 0.5717 | 8.717 | 0.1523 | 0.3683 | 0.1972 | 0.2381 | 0.2461 |
| **500** | **100** | 8.087 | 0.2931 | 0.4781 | 0.5639 | 12.51 | 0.7675 | 0.5751 | 0.3069 | 0.3274 | 0.3524 |
|  | **70** | 4.341 | 0.1894 | 0.2649 | 0.4177 | 8.964 | 0.4061 | 0.3973 | 0.2098 | 0.2467 | 0.2593 |
|  | **50** | 2.980 | 0.1732 | 0.2396 | 0.3831 | 6.748 | 0.3762 | 0.3531 | 0.1993 | 0.1934 | 0.1941 |
|  | **30** | 1.709 | 0.1214 | 0.1890 | 0.3146 | 4.284 | 0.1535 | 0.1693 | 0.1538 | 0.1647 | 0.1527 |

**Table S4**. Number of differential metabolites identified by different methods.

| **Different Methods** | **Original Data** | **Original Data with 5% outliers** | **Original Data with 10% outliers** | **Original Data with 15% outliers** |
| --- | --- | --- | --- | --- |
| **Proposed** | 37 | 37 | 37 | 37 |
| ***t*-test** | 54 | 24 | 11 | 31 |
| **Wilcoxon** | 58 | 53 | 48 | 46 |
| **KW** | 58 | 53 | 48 | 47 |
| **BRIDGE** | 65 | 59 | 53 | 48 |
| **FCROS** | 23 | 22 | 22 | 24 |
| **Limma** | 36 | 36 | 36 | 36 |
| **Ebarrays** | 34 | 23 | 34 | 25 |
| **SAM** | 52 | 36 | 34 | 41 |
| **CVP** | 36 | 32 | 25 | 20 |

Note: Differential metabolites were identified from the experimental dataset both in the absence and presence of (artificially imputed) outliers.
