# Supplementary material for: Robust volcano plot: identification of differential metabolites in the presence of outliers
Source: BMC Bioinformatics. 2018 Apr 11;19:128. doi: 10.1186/s12859-018-2117-2 (PMC5896081; doi:10.1186/s12859-018-2117-2)
Supplement: Supplementary file 2 — Performance evaluation of the proposed technique compared to other techniques using ROC curves and MER and AUC values for the artificial datasets in the absence and presence of outliers. Figure S1. Performance evaluation using ROC curves for different differential metabolite identification techniques (a) in the absence of outliers, (b) with 5% outliers, (c) with 10% outliers, (d) with 15% outliers, (e) with 20% outliers, and (f) with 25% outliers. Figure S2. Performance evaluation using box plots of 500 MERs for different differential metabolite identification techniques (a) in the absence of outliers, (b) with 5% outliers, (c) with 10% outliers, (d) with 15% outliers, (e) with 20% outliers, and (f) with 25% outliers. Figure S3. Performance evaluation using box plots of 500 AUC values for different differential metabolite identification techniques (a) in the absence of outliers, (b) with 5% outliers, (c) with 10% outliers, (d) with 15% outliers, (e) with 20% outliers, and (f) 25% outliers. Figure S4. Performance evaluation using Venn diagrams for the number of differential metabolites identified by different differential metabolite identification methods for the experimental dataset. (DOC 6677 kb) [file 12859_2018_2117_MOESM2_ESM.doc]

# Additional file 2:

**Figure S1.** Performance evaluation using ROC curves for different differential metabolite identification techniques (a) in the absence of outliers, (b) with 5% outliers, (c) with 10% outliers, (d) with 15% outliers, (e) with 20% outliers, and (f) with 25% outliers.


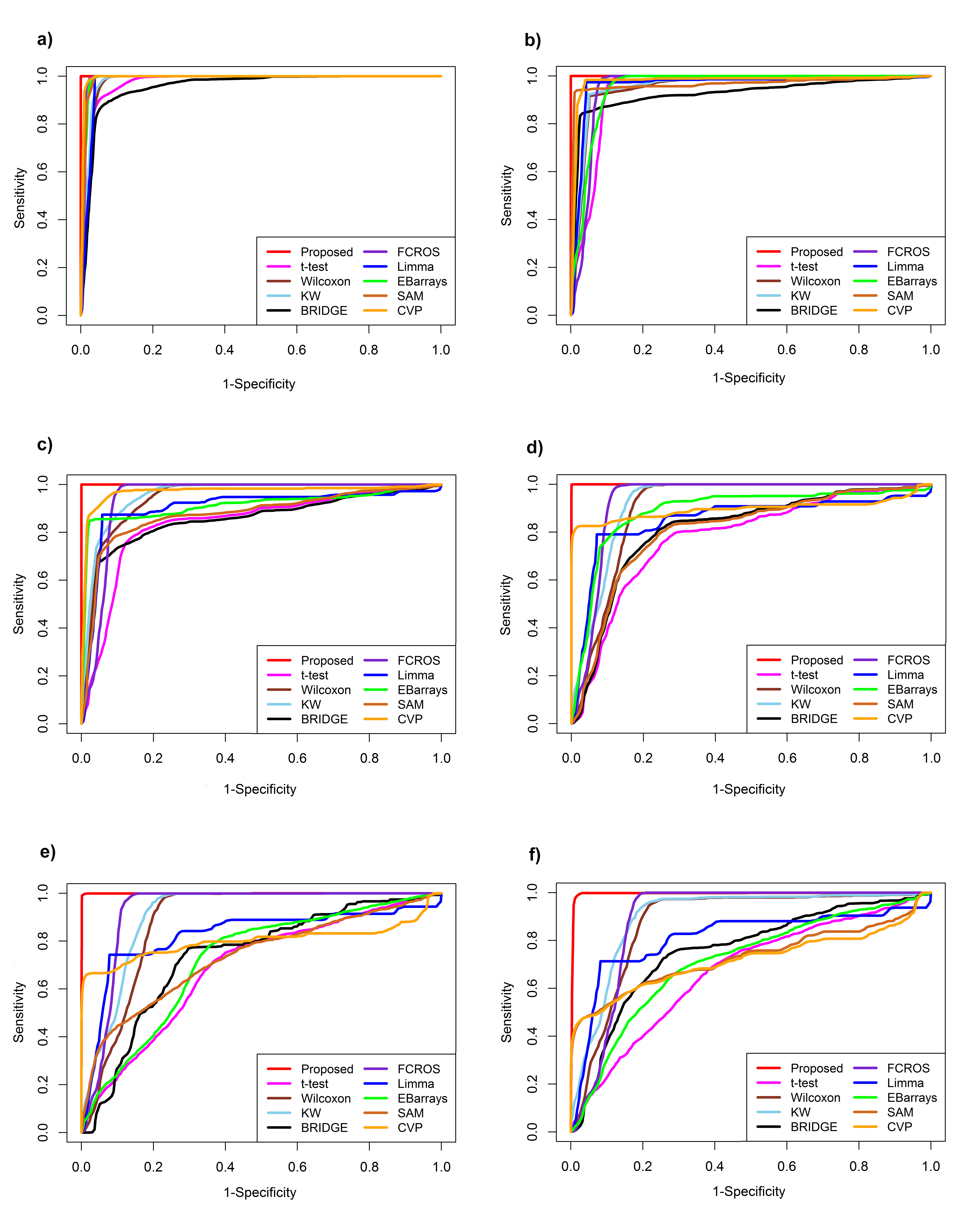


**Figure S2.** Performance evaluation using box plots of 500 MERs for different differential metabolite identification techniques (a) in the absence of outliers, (b) with 5% outliers, (c) with 10% outliers, (d) with 15% outliers, (e) with 20% outliers, and (f) with 25% outliers.


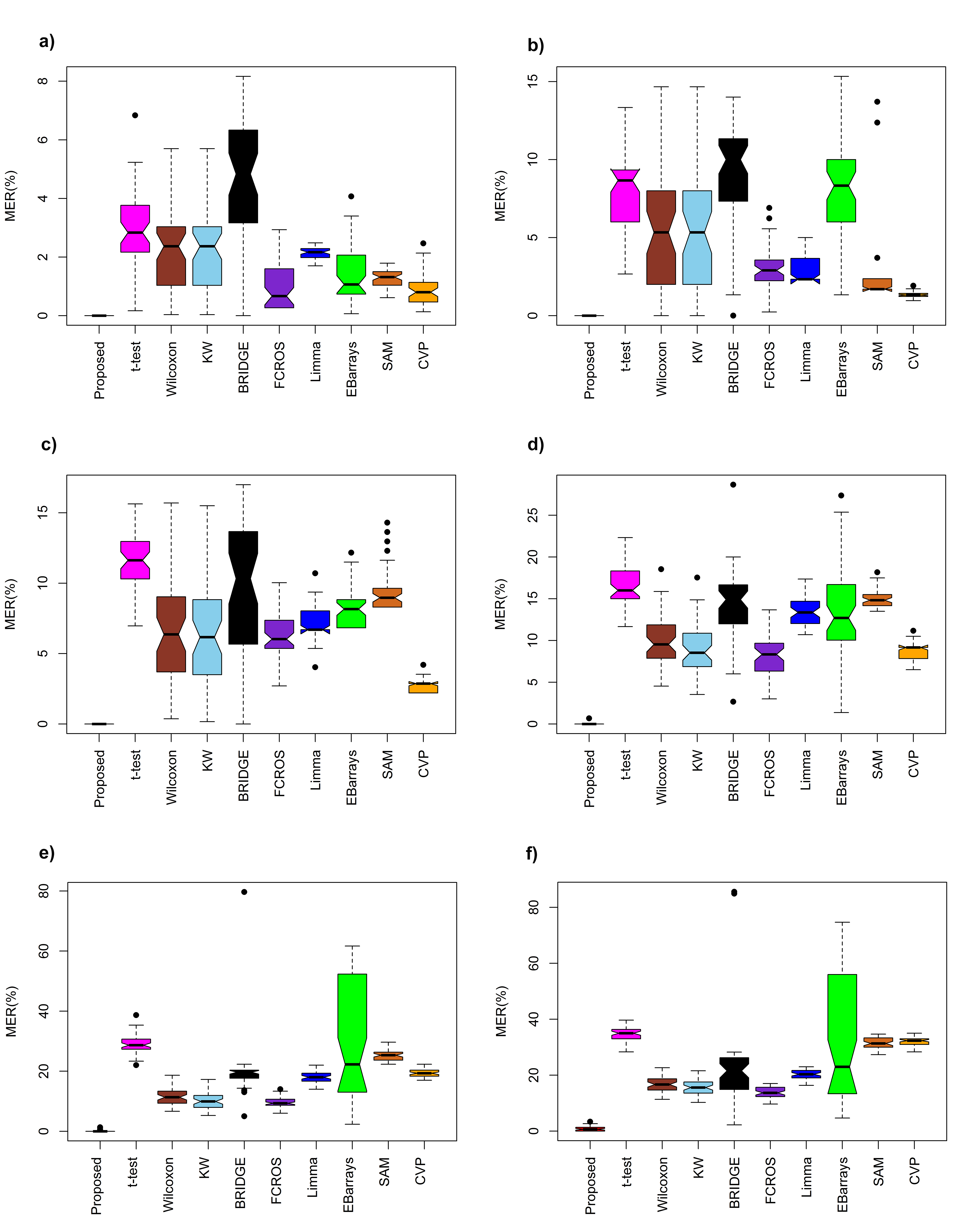


**Figure S3**. Performance evaluation using box plots of 500 AUC values for different differential metabolite identification techniques (a) in the absence of outliers, (b) with 5% outliers, (c) with 10% outliers, (d) with 15% outliers, (e) with 20% outliers, and (f) 25% outliers.


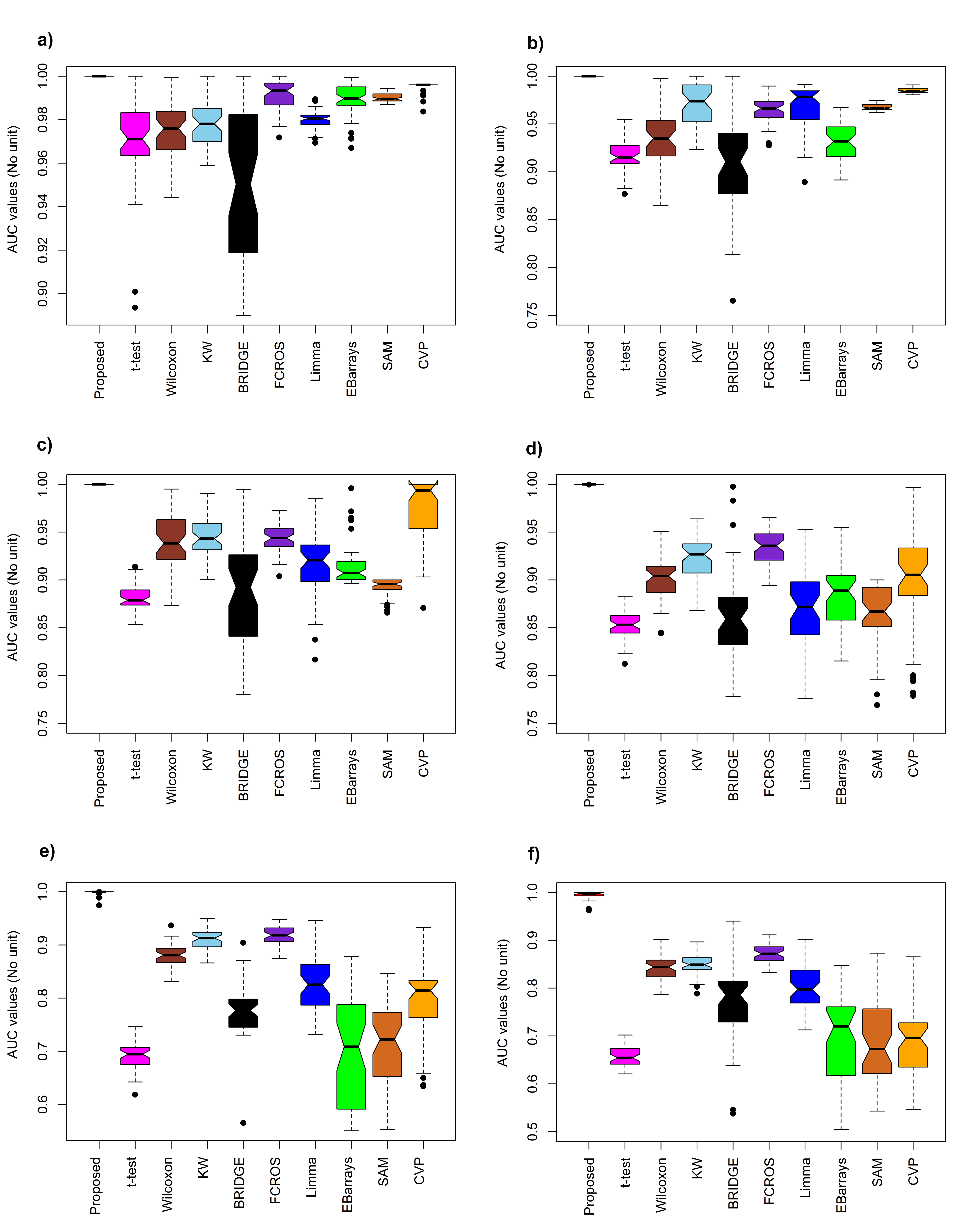


**Figure S4.** Performance evaluation using Venn diagrams for the number of differential metabolites identified by different differential metabolite identification methods for the experimental dataset.

**
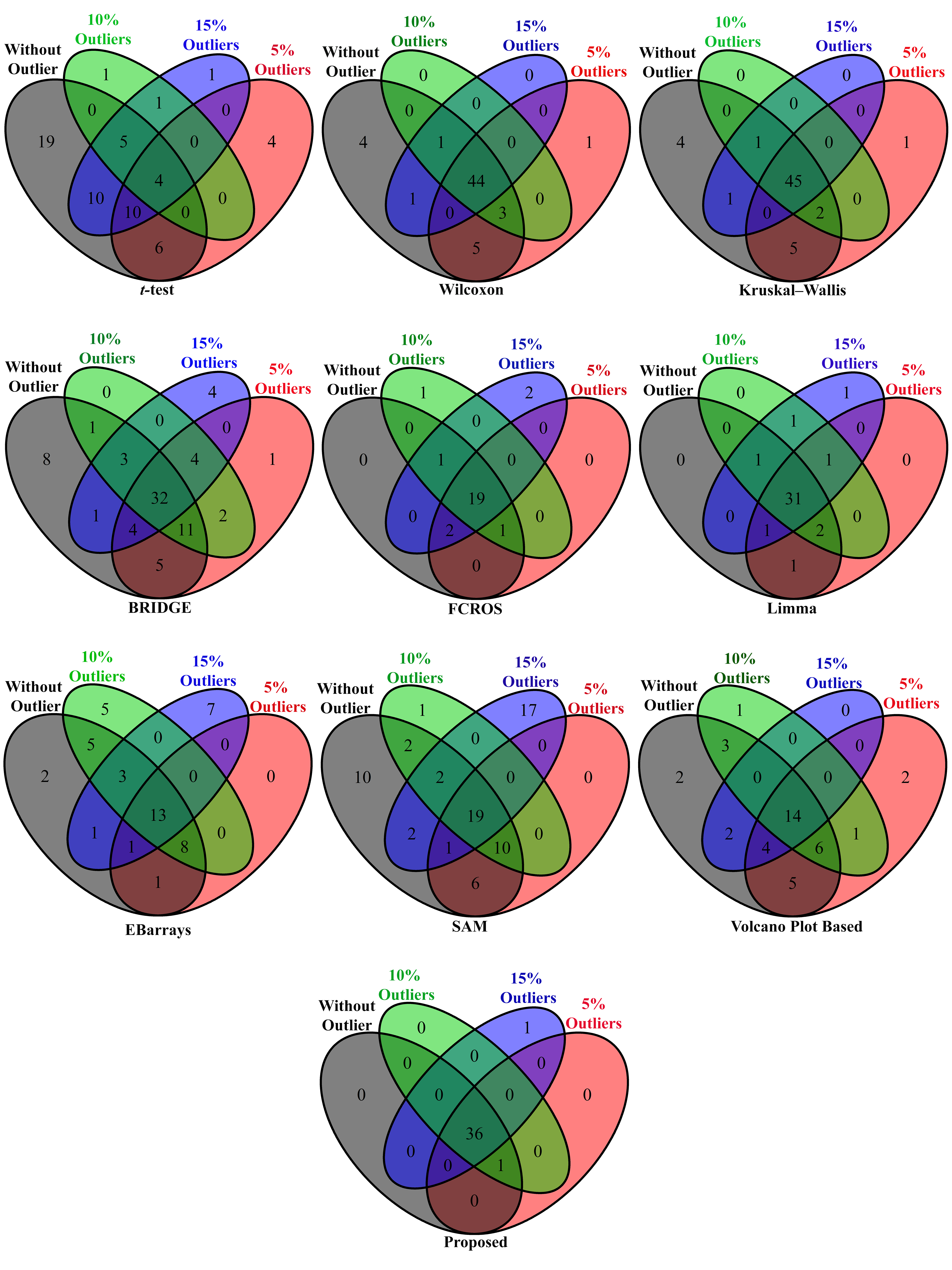
**
